# Species-specific blood–brain barrier permeability in amphibians

**DOI:** 10.1186/s12915-025-02145-7

**Published:** 2025-02-11

**Authors:** Sophie Antesberger, Beate Stiening, Michael Forsthofer, Alberto Joven Araus, Elif Eroglu, Jonas Huber, Martin Heß, Hans Straka, Rosario Sanchez-Gonzalez

**Affiliations:** 1 Graduate School of Systemic Neurosciences, Ludwig-Maximilians-University Munich, Großhaderner Str. 2, 82152 Planegg, Germany; 2 Faculty of Biology, Ludwig-Maximilians-University Munich, Großhaderner Str. 2, 82152 Planegg, Germany; 3 Department of Neuroscience, University of Sussex, Brighton and Hove, UK; 4 Department of Cell and Molecular Biology, Karolinska Institutet, Stockholm, Sweden

**Keywords:** *Xenopus laevis*, *Ambystoma mexicanum*, *Pleurodeles waltl*, Transcytosis, Paracellular transport

## Abstract

**Background:**

The blood–brain barrier (BBB) is a semipermeable interface that prevents the non-selective transport into the central nervous system. It controls the delivery of macromolecules fueling the brain metabolism and the immunological surveillance. The BBB permeability is locally regulated depending on the physiological requirements, maintaining the tissue homeostasis and influencing pathological conditions. Given its relevance in vertebrate CNS, it is surprising that little is known about the BBB in Amphibians, some of which are capable of adult CNS regeneration.

**Results:**

The BBB size threshold of the anuran *Xenopus laevis* (African clawed toad), as well as two urodele species, *Ambystoma mexicanum* (axolotl) and *Pleurodeles waltl* (Iberian ribbed newt), was evaluated under physiological conditions through the use of synthetic tracers. We detected important differences between the analyzed species. *Xenopus* exhibited a BBB with characteristics more similar to those observed in mammals, whereas the BBB of axolotl was found to be permeable to the 1 kDa tracer. The permeability of the 1 kDa tracer measured in *Pleurodeles* showed values in between axolotl and *Xenopus vesseks*. We confirmed that these differences are species-specific and not related to metamorphosis. In line with these results, the tight junction protein Claudin-5 was absent in axolotl, intermediate in *Pleurodeles* and showed full-coverage in *Xenopus* vessels. Interestingly, electron microscopy analysis and the retention pattern of the larger tracers (3 and 70 kDa) demonstrated that axolotl endothelial cells exhibit higher rates of macropinocytosis, a non-regulated type of transcellular transport.

**Conclusions:**

Our study demonstrated that, under physiological conditions, the blood–brain barrier exhibited species-specific variations, including permeability threshold, blood vessel coverage, and macropinocytosis rate. Future studies are needed to test whether the higher permeability observed in salamanders could have metabolic and immunological consequences contributing to their remarkable regenerative capacity.

**Supplementary Information:**

The online version contains supplementary material available at 10.1186/s12915-025-02145-7.

## Background

The protection of the vertebrate brain from physical disruptions of external and internal barriers is a prerequisite for the functionality of this organ. While the skull, several meningeal layers and the cerebrospinal fluid form the boundary with the external environment, the brain parenchyma is separated internally from the vascular system by a tight blood–brain barrier (BBB), which ensures a biochemically controlled homeostatic environment [1]. The BBB forms early during development and thereafter regulates the molecular and cellular trafficking between the vascular compartment and the neural tissue [2]. The BBB is not a static barrier but rather a highly plastic interface that is able to prevent a non-selective molecular exchange. However, with the potential to alter the permeability depending on physiological requirements. The regulated traffic across the BBB is constrained by the energy consumption of the brain that requires a constant blood supply to provide molecules for fueling the energetic demands for neuronal function [3, 4]. In addition, the brain is an immune-privileged organ, where trafficking of immune cells and antibodies is severely restricted [5]. Only blood-derived immune cells involved in immunological surveillance are able to cross under normal physiological conditions. However, upon signs of damage or infection the BBB permeability can rapidly change to allow an extravasation of peripheral immune cells into the brain [5]. The functionality of the BBB relies on a complex structure established by both cellular and extra-cellular components that tightly control blood–brain trafficking. Dysfunction of the BBB, such as slight changes in permeability, can have devastating consequences including initiation and/or progression of neurological diseases such as epilepsy [6], cognitive decline [7], multiple sclerosis, stroke or Alzheimer’s disease [8]. The composition of the BBB varies across taxa, while most of vertebrate barriers possess the vascular endothelium, the invertebrate ones generally involve glial cells [9]. The structure of the mammalian BBB is well known, with endothelial cells (ECs), mural cells (including pericytes and vascular smooth muscle cells) and astrocytic endfeet interacting together to establish the so-called neurovascular unit or neurovascular complex [10]. ECs constitute the inner lining of the vascular wall and represent the first barrier for blood-borne molecules. In contrast to ECs in the blood vessels of the body, ECs in the brain vascular system lack fenestrations [11] and have a restricted transcellular trafficking [12], thereby contributing to the selective permeability of the BBB. In addition, the paracellular diffusion is severely reduced through the presence of tight and adherent junctions [13]. Despite the apparent morphological homogeneity, different subpopulations of ECs might allow regionally different regulatory schemes of the BBB [14]. On the side of the brain, astrocytic endfeet cover the entire outer surface of the blood vessels, representing the last barrier between the vasculature and the brain parenchyma. Astrocytes are critical for the integrity of the BBB in mammalian species [15] and represent a major metabolic checkpoint [16].

The relevance of the BBB to protect and maintain the homeostatic, nutritive and immune-privileged status of the central nervous system (CNS) raises the question of how vertebrates, such as amphibians, establish a functional barrier in the absence of one of the main regulatory cell types, the astrocytes. Instead, the amphibian brain possesses ependymoglial cells [17] a multifaceted population capable of exerting several functions. Similar to the mammalian astrocytes, ependymoglial endfeet have been described to contact the blood vessels in salamanders [18, 19], but its potential role in regulating the BBB remains obscure. Surprisingly, and despite amphibians emerging as a primary animal model for biological research, little is known about the BBB permeability [9] and if they have a functional barrier comparable in tightness to that in mammalian species. We hypothesized that the BBB permeability in Amphibians might vary depending on the species, ultimately influencing their regenerative potential. Here, we evaluated the permeability of the brain vascular system in an anuran species with a CNS regenerative capacity restricted to larval stages, *Xenopus laevis* (African clawed toad), as well as in two urodele species which retain CNS regenerative abilities during adulthood, *Ambystoma mexicanum* (axolotl) and *Pleurodeles waltl* (Iberian ribbed newt). Since the mammalian BBB is impermeable for synthetic dyes with a molecular weight ≥ 1 kDa [20], different tracers with 1, 3 and 70 kDa were transcardially injected [21] into the vascular system and the retention of the dyes in the blood vessels or the leakage into the brain parenchyma was assessed. We demonstrated that under physiological conditions amphibians displayed different permeability thresholds depending on the species, together with differences in tight junction’s coverage. Like mammals, the *Xenopus* BBB is characterized by the presence of Claudin-5 in all blood vessels and it is impermeable to all analyzed tracers. The brain parenchyma in *Pleurodeles* contains blood vessels with and without Claudin-5 protein, showing some leakiness of the smallest dye (1 kDa) into the brain parenchyma. Axolotl showed the most permeable BBB, coinciding with the total lack of Claudin-5 expression in the brain. Moreover, axolotl ECs exhibit higher rates of macropinocytosis, a non-regulated type of transcellular transport.

## Results

### Labeling of the vascular system in the *Xenopus* and axolotl brains

**Fig. 1:**
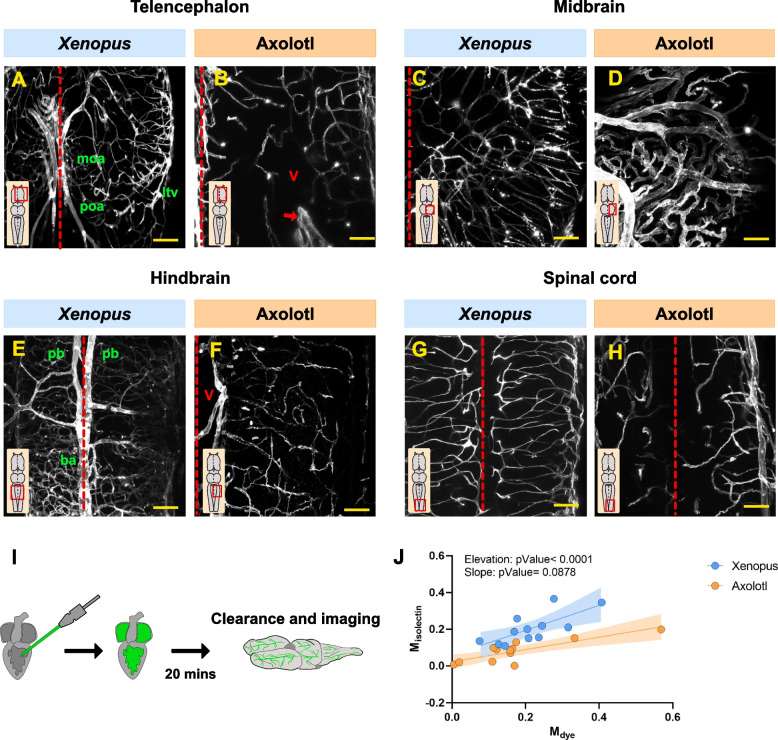
Size-selective permeability analysis in the axolotl and *Xenopus* BBB. **A-H** Confocal images illustrating the *Xenopus* (**A**, **C**, **E**, **G**) and axolotl (**B**, **D**, **F**, **H**) vasculature labelled with isolectin. Red dashed lines delineate the brain midline. The arrow indicates the choroid plexus. All images are full z-projections of a confocal stack from cleared brains. Insets indicate the area of the brain. Scale bars = 100 µm. **I** Scheme of the experimental workflow to analyze the BBB permeability. **J** Scatter plot depicting the Manders’ coefficients obtained after injecting the 1, 3 and 70 kDa tracers in both axolotl and *Xenopus*. Data points were color-coded according to the species (blue: *Xenopus*; orange: axolotl). Each data point represents one animal and the statistical analysis was based on simple linear regression (Slope: pValue = 0.0878; Elevation: *p*Value < 0.0001). Abbreviations: ba: basilar artery; ltv: lateral telencephalic vein; moa: medial olfactory artery; pb: posterior branch of cerebral carotid; poa: preoptic artery; V: ventricle

The brain vasculature was labelled by transcardially injecting Isolectin GS-IB4 from *Griffonia simplicifolia*, which specifically binds to glycoconjugates in the basement membrane [22]. Our method successfully labeled amphibian vascular systems including the fine capillary networks (Fig. 1A-I; Additional file 1: Fig. S1A). A comparison with previously published vascular anatomy of *Xenopus* [23] allowed us to identify several of the main arteries and veins irrigating the *Xenopus* brain (Fig. 1A, E), demonstrating the reproducibility of our method.

### Size permeability analysis

The ability to cross the BBB is determined by physicochemical properties such as size, charge, polarity and receptor/carrier recognition [24]. In this study we used specific synthetic tracers, the permeability of which is only determined by their molecular size. A mature and healthy mammalian BBB is impermeable to dextrans of 1 kDa (comparable to small peptides) [15], 3 kDa (comparable to small proteins) and 70 kDa (comparable to albumin, one of the biggest plasma proteins) [25]. The permeability size threshold was assessed in both *Xenopus* and axolotl by transcardially injecting of either 1, 3 or 70 kDa tracers in combination with isolectin (marker for blood vessels). The co-occurrence coefficients of the two fluorescence signals (selected tracer and isolectin) provided evidence of the permeability of the BBB to the analyzed tracers. A high value indicated low permeability (retained in the blood vessels), while a low coefficient indicated high permeability. The co-occurrence coefficients of all the injected animals (1 kDa, 3 kDa and 70 kDa in both *Xenopus* and axolotl) were plotted and an unbiased analysis was performed. Our data were color-coded according to the tracer molecular size (Additional file 1: Fig. S1B) or to the species (Fig. 1J). Surprisingly, no significant differences were observed when the tracer sizes were plotted (Additional file 1: Fig. S1B); however, the coefficients were significantly segregated by species (Fig. 1J). These data suggested that the analyzed amphibian species have different permeability thresholds. Specifically, the lower Manders' coefficients in the axolotl samples indicated that some of the dyes were not localized in the vasculature and might systemically cross the BBB. To validate this hypothesis, further analysis of each of the injected tracer was performed.

### Permeability of large molecules in the Amphibian brain

**Fig. 2:**
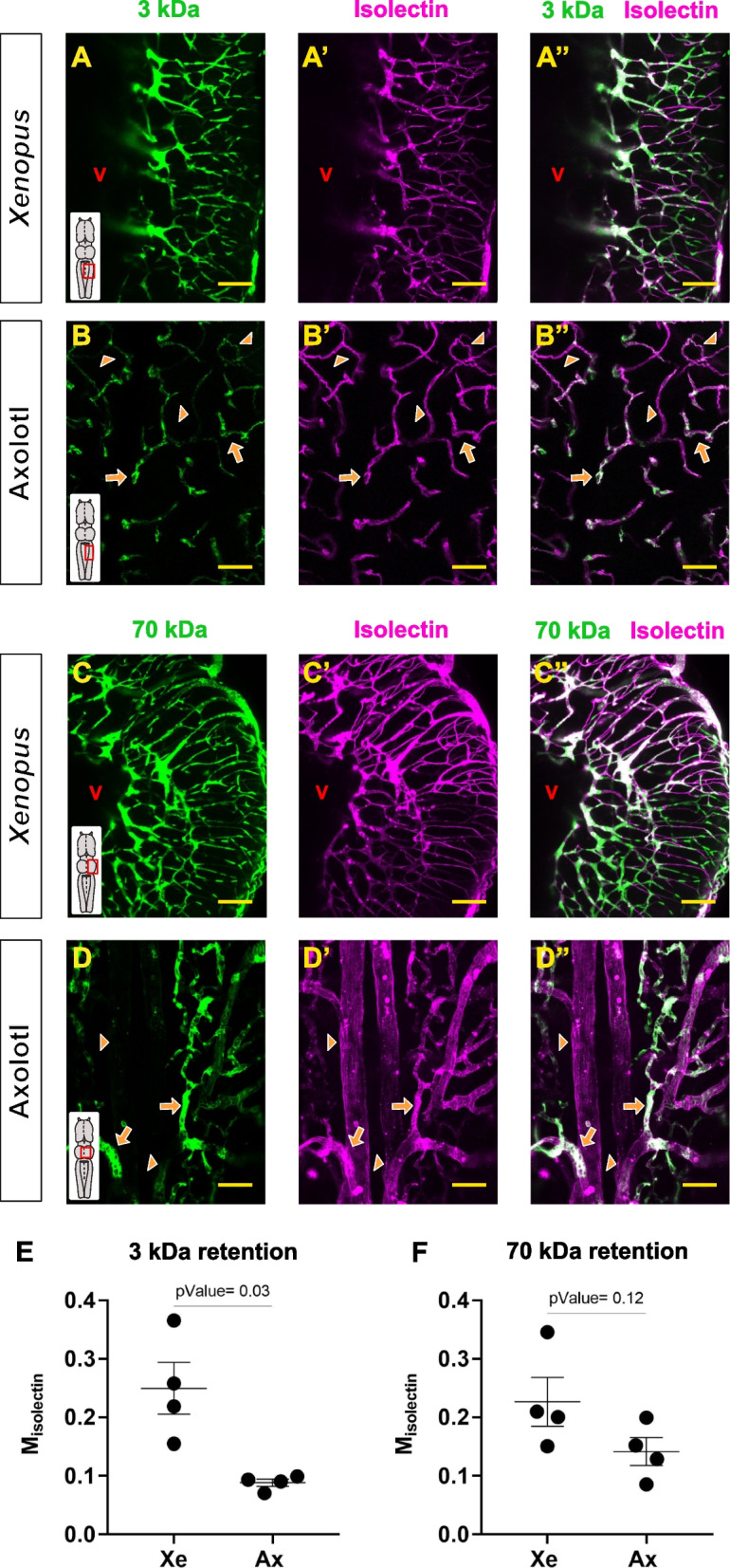
Analysis of the 3 and 70 kDa tracer permeability in the axolotl and *Xenopus* BBB. **A-D’’** Confocal micrographs of cleared whole-mount *Xenopus* (**A-A’’**, hindbrain; **C–C’’**, midbrain) and axolotl (**B-B’’**, hindbrain; **D-D’’**, midbrain) CNS injected with the 3 kDa (**A**-**B’’**) or the 70 kDa (**C**-**D’’**) tracer together with isolectin. Low retention areas (3 kDa: **B-B’’**; 70 kDa: **D**-**D’’**) were indicated with orange arrowheads and high retention areas with orange arrows. All images are full z-projections of a confocal stack. Insets indicate the area of the brain. Scale bars = 100 µm. (**E**, **F**) Graphs depicting the M_isolectin_ coefficients after 3 kDa (**E**) and 70 kDa (**F**) injections in *Xenopus* and axolotl. Each data point represents one animal. Data are shown as mean ± SEM and statistical analysis is based on Welch´s t-test (**E**; p-Value = 0.03) and Student´s t-test (**F;** p-Value = 0.12). Abbreviations: Ax: axolotl; V: ventricle; Xe: *Xenopus*

**Fig. 3:**
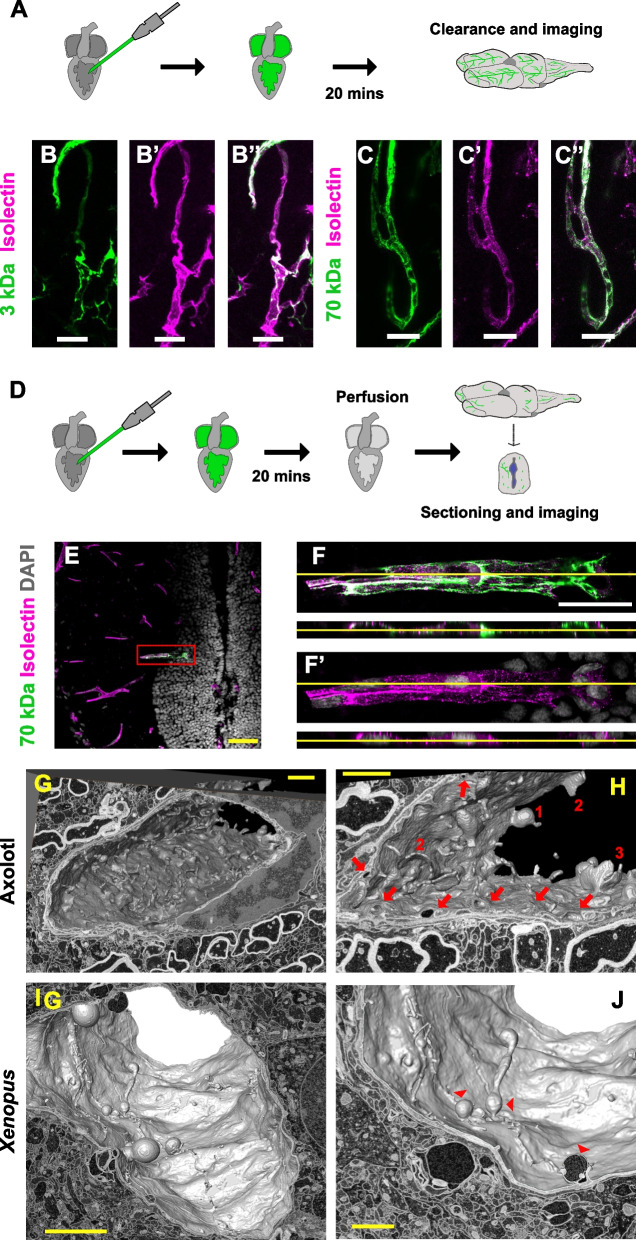
Analysis of tracer uptake by the axolotl ECs. **A** Scheme of the experimental design analyzing the permeability of the larger tracers. **B-C’’** Images illustrating the colocalization of the 3 kDa (**B-B’’**) and 70 kDa (**C–C’’**) tracers with isolectin^+^ blood vessels. **D** Scheme representing the experimental set-up utilized to assess the tracers EC uptake. **E** Confocal image of a transversal section of the axolotl hindbrain showing the location of the 70 kDa tracer after perfusion. **F**, **F'** are magnifications of the boxed area in (**E**). The orthogonal projections illustrated the localization of the 70 kDa tracers inside isolectin^+^ EC. All images are full z-projections of a confocal stack. **G, H** Internal axolotl EC surface (width ca. 8 × 12 µm, depth 16.4 µm). The surrounding EM plane shows a cell nucleus (right) and numerous profiles of neurites. Type of ruffles (1: blebs; 2: planar folds; 3: cup-shaped). Red arrows: macropinosomes. (**I, J**) EM reconstruction of a *Xenopus* EC luminal surface (width ca. 14 × 24 µm, depth 16 µm). Red arrowheads: extracellular vesicles. Scale bars in B, B’, B’’, C, C’, C’’ = 20 µm; E, F, F’’ = 100 µm; G, H, J = 2 µm; I = 5 µm

Our analysis suggested that under physiological conditions the axolotl BBB might be more permeable than *Xenopus*. Therefore, we analyzed the permeability of the larger tracers (3 and 70 kDa) in cleared *Xenopus* and axolotl brains side by side. Both the 3 and the 70 kDa tracers were fully retained in the *Xenopus* blood vessels (Fig. 2A-A’’, C–C’’), in line with the higher Manders’ coefficients (Fig. 2E, F). In contrast, the axolotl BBB displayed a patchy labelling pattern with high and low retention areas for both tracers (Fig. 2B-B’’, D-D’’). In the high retention areas, isolectin-positive blood vessels were completely filled with dyes (Fig. 2, arrows), while the low retention areas showed little to no tracers in the vasculature (Fig. 2, arrowheads). The 3 kDa dye was significantly more permeable between axolotl and *Xenopus*, while the 70 kDa dye only showed a non-significant trend (Fig. 2E, F). Despite these results suggesting changes in local permeability, we did not observe any tracer located inside of the axolotl brain parenchyma (Fig. 2B-B’’, D-D’’). Rather, in some instances dyes appeared to be located intracellularly in the vasculature compartment. The morphology and density of these isolectin^+^ cells indicate that some ECs might have uptake the larger tracers (Fig. 3 A-C’’). We therefore hypothesized that the patchy retention pattern might be at least partially due to increased EC endocytosis. To explore this hypothesis, we injected the 70 kDa tracer together with isolectin and after 20 min survival, the animals were perfused in order to wash out any traces of blood and circulating dye (Fig. 3D). The axolotl brains were then sectioned and imaged. With this protocol, any remaining tracer must have been taken in by the ECs or by other resident brain cells, allowing us to assess both endocytosis and transport to the parenchyma. Indeed, we observed isolectin^+^ ECs that incorporated the 70 kDa tracer intracellularly (Fig. 3E-F’), validating the local changes in permeability shown in the whole-mounts. However, we still did not observe any signs of leakage or complete transport across the BBB to the brain parenchyma (Fig. 3E). Irrespective of the type of transcellular transport (transcytosis), the general underlying mechanism is rather similar. First, macromolecules are endocytosed by vesicles that enter the EC endosomal network. Sorting mechanisms will determine whether a given molecule will be recycled back to the blood, transport to the brain parenchyma or to lysosomes for degradation [26]. Considering the transcytosis kinetics, the 20 min survival protocol might not be long enough to detect active transport. So next, we increased the survival time to 60 min and analyzed the co-occurrence coefficients in whole-mount preparations (Additional file 1: Fig. S2). Surprisingly, the low/high retention pattern was still present and no obvious signs of transport or leakage to the neural tissue were observed in the axolotl brain (Additional file 1: Fig. S2B-B’). The longer survival protocol didn’t substantially change the retention phenotype observed in the *Xenopus* brain either; however, areas with low tracer retention started to be more evident (Additional file 1: Fig. S2C-C’). In addition, the co-occurrence coefficients of the 70 kDa tracer remained constant irrespectively of the survival time or the species (Additional file 1: Fig. S2D), suggesting that the endocytosis occurs selectively in a subset of brain vessels. Taking together, both axolotl and *Xenopus* BBBs seem to be impermeable to larger synthetic tracers. However, our experiments also revealed that axolotl ECs might activate selective endocytosis events at a higher rate than *Xenopus*.

### Electron microscopy analysis of the axolotl and *Xenopus* endothelial cells

The higher rate of tracer uptake by the axolotl ECs indicated not only differences in the regulation of the BBB permeability, but might also have metabolic, immunological and physiological consequences. To validate this finding and study which type of endocytic pathway might be specifically activated, we performed electron microscopy analysis in both axolotl and *Xenopus*. The 3D reconstruction of the EC lumen surface showed major differences between the two species. Axolotl ECs displayed a high density of large membrane protrusions or ruffles (Fig. 3G, H) in comparison to *Xenopus* (Fig. 3I, J). Different types of ruffles could be identified according to shape and size: planar folds (lamellipodia-like), blebs (large membrane protrusion) and cup-shaped ruffles. We also observed a high number of vesicles in different stages of formation that varied in size from medium (~ 0.25 µm) to large (~ 1.4 µm). While the larger vesicles appeared to be formed by large membrane protrusions (Additional file 1: Fig. S3A), some of the medium-size vesicles were formed by membrane invaginations (Additional file 1: Fig. S3B). In contrast to the capillary lumen (and not fully closed engulfing structures) the observed large vesicles contained flaky electron dense material from the time prior to fixation. The *Xenopus* EC lumen surface exhibited a smoother appearance in comparison with the axolotl data (Fig. 3I). The number of membrane protrusions was extremely reduced, and instead, the *Xenopus* ECs formed large vesicles (diameter ca. 1–2 µm) protruding towards the lumen (Fig. 3J; Additional file 1: Fig. S3C). These multichambered extracellular vesicles also contained flaky electron dense material. Similar to axolotl, small to medium-size (~ 0.1–0.25 µm) intracellular vesicles were observed, with low density of larger vesicles (Additional file 1: Fig. S3D).

### Leakiness of the 1 kDa tracer in the axolotl brain

**Fig. 4:**
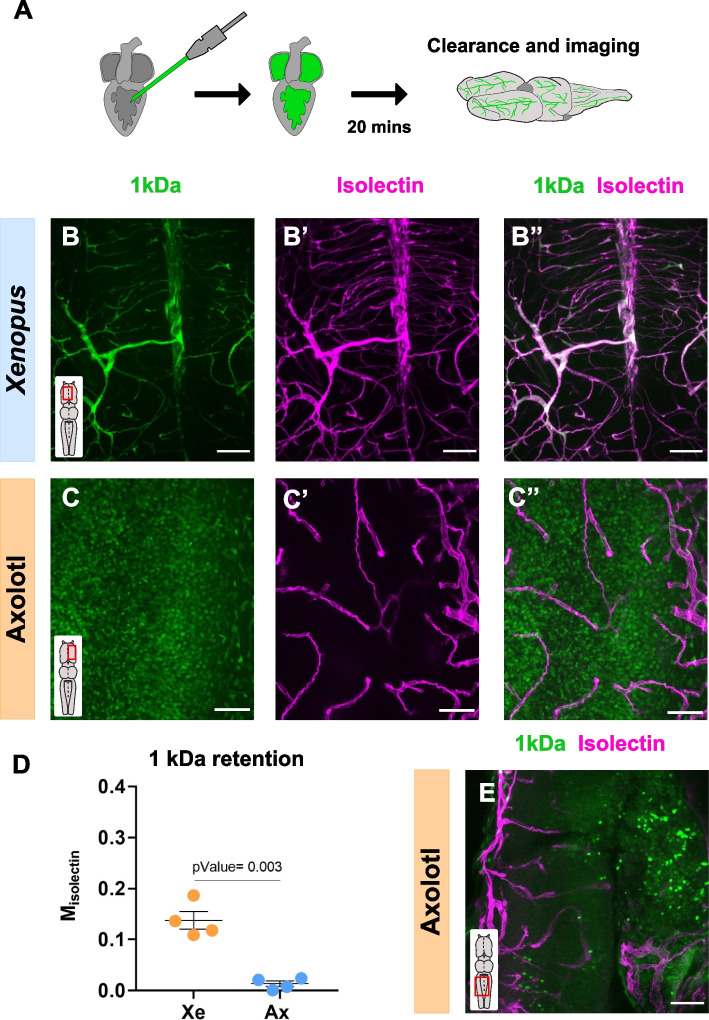
Analysis of the 1 kDa tracer permeability in the Amphibian CNS. **A** Experimental design to analyze the BBB permeability. **B-C’’** Micrographs of cleared whole-mounts *Xenopus* (**B-B’’**) and axolotl (**C–C’’**) telencephalon showing the distribution of the injected 1 kDa tracer. The vasculature was labeled using isolectin. **D** Graph indicating the Manders’ coefficients in the *Xenopus* (blue dots) and axolotl (orange dots) BBB. Each data point represents one animal. Data are shown as mean ± SEM and statistical analysis is based on Welch´s t-test (p-Value = 0.003). **E** Micrograph of a cleared whole-mount axolotl hindbrain injected with the 1 kDa tracer and isolectin after 10 min survival. Inset indicate the area of the brain. All images are full z-projections of a confocal stack. Scale bars = 100 µm. Abbreviations: Ax: axolotl; Xe: *Xenopus*

The majority of the blood-borne molecules, with the exception of small lipophilic molecules (under 400 Da), must cross the BBB using active transcytosis mechanisms [24]. Cadaverine (used in this study) and other 1 kDa tracers have been extensively used to assess BBB leakiness in pathological conditions [15, 16], since it is one of the smallest tracers that has been described to be retained in the blood vessels. Pre-metamorphic *Xenopus* brain showed the 1 kDa tracer colocalizing inside the isolectin^+^ blood vessels (Fig. 4A, B-B’’, D; Additional file 1: Fig. S4A), recapitulating the permeability threshold observed in physiological conditions in *Xenopus* [27], mammals [20] and zebrafish [28]. On the other hand, we found that the axolotl BBB was fully permeable to the 1 kDa tracer, labelling brain parenchyma cells (Fig. 4C-C’’; Additional file 1: Fig. S4C-D’’) and exhibiting Manders’ coefficients close to 0 (Fig. 4D; Additional file 1: Fig. S4B). In physiological conditions, paracellular transport is severely restricted and the majority of the trafficking is achieved by active transcytosis. However, the short survival time used in our protocol (20 min) and the almost complete lack of colocalization with the utilized EC marker (Fig. 4C-C’’), supported paracellular passive transport rather than transcytosis as the underlying mechanism. In order to validate this hypothesis, the survival time after the injection was further reduced to 10 min (Fig. 4E). As predicted, we still observed leakage of the 1 kDa tracer into the axolotl brain parenchyma, although to a smaller extent. The fast and systemic leakage of the smallest tracer strongly suggests that molecules up to 1 kDa might passively cross the axolotl BBB via paracellular transport.

Considering the observed differences in *Xenopus* and axolotl BBB permeability, an additional permeability assay of the 1 kDa tracer was conducted in *Xenopus* at different developmental stages. As expected, before the establishment of the BBB [27] (Stage 49) the tracer leaked to the brain parenchyma (Additional file 1: Fig. S4E, E’), albeit to a lesser extent than the axolotl case (Additional file 1: Fig. S4D-D’’). In line with our previous data, post-metamorphic toads fully retained the tracer in the vasculature (Additional file 1: Fig. S4F, F’).

### Partial leakage in the pre- and post-metamorphic salamander *Pleurodeles waltl*

**Fig. 5:**
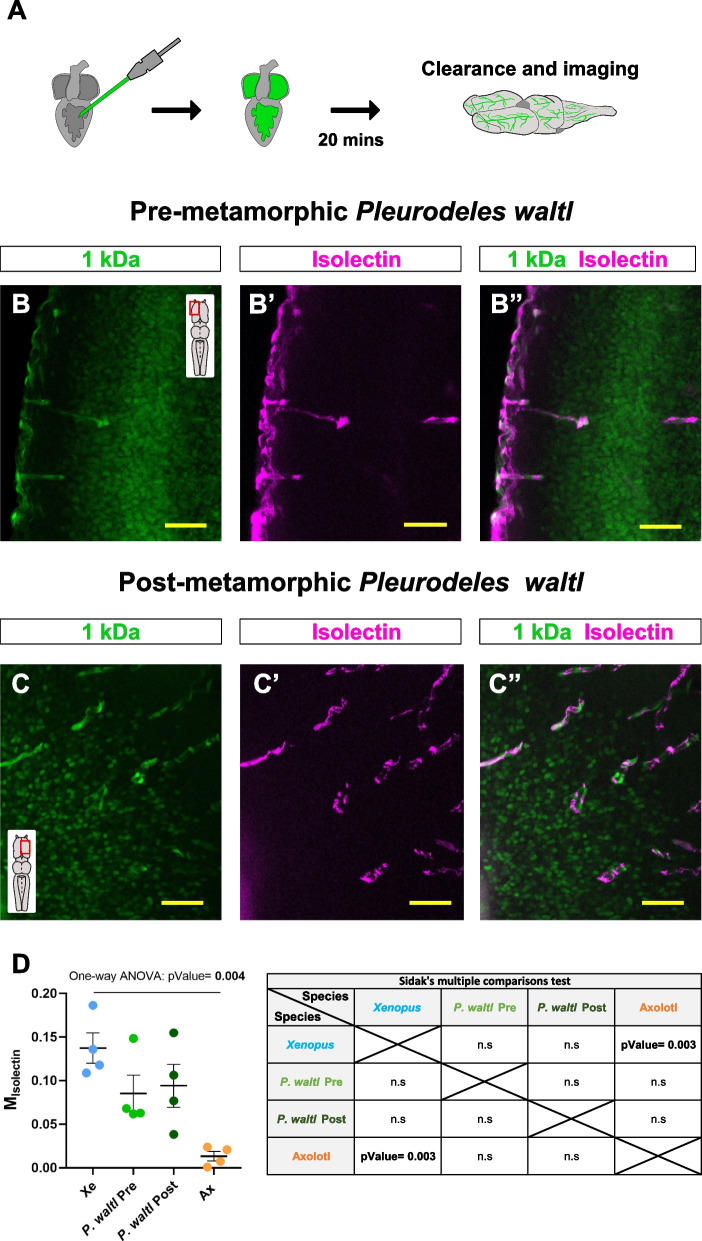
Analysis of the permeability threshold in pre- and post-metamorphic *Pleurodeles*. **A** Scheme of the experimental workflow. **B**-**C’’** Confocal images of cleared whole-mount telencephala in pre- (**B-B’’**) and post-metamorphic (**C–C’’**) *Pleurodeles* after co-injecting the 1 kDa tracer with isolectin. All images are full z-projections of a confocal stack. Insets indicate the area of the brain. Scale bars = 50 µm. **D** Dot-plot indicating the Manders’ coefficient for the 1 kDa tracer in *Xenopus* (blue dots), *Pleurodeles* pre-metamorphic (light green dots), *Pleurodeles* post-metamorphic (dark green dots) and axolotl (orange dots). Each data point represents one animal. Data are shown as mean ± SEM and statistical analysis is based on One-way ANOVA (pValue = 0.004) and Sidak’s multiple comparisons test (Xe vs. Pleurodeles Pre: pValue = 0.357; Xe vs. Pleurodeles Post: *p*Value = 0.555; Xe vs. Ax: *p*Value = 0.003; Pleurodeles Pre vs. Pleurodeles Post: pValue > 0.9999; Pleurodeles Pre vs. Ax: pValue = 0.102; Pleurodeles Post vs. Ax: *p*Value = 0.055). Abbreviations: Ax: axolotl; n.s: not significant; Pre: pre-metamorphic; Post: Post-metamorphic; Xe: *Xenopus*

The size permeability assay indicated that the BBB permeability threshold may vary depending on the Amphibian species. Unlike the rest of the analyzed vertebrates, young axolotl showed passive leakiness of the smaller tracer (1 kDa), which is classically considered pathological. However, axolotl do not spontaneously undergo through metamorphosis, retaining larval traits during adulthood (paedomorphosis). To test whether the leakage observed in axolotl was a larval/paedomorphic trait, we injected a combination of 1 kDa and isolectin in both pre- and post-metamorphic *Pleurodeles* (Fig. 5A). Both stages showed comparable retention patterns in the blood vessels and no significant differences were detected (Fig. 5B-D). Strikingly, the permeability of the 1 kDa tracer measured in *Pleurodeles* showed values in between the axolotl and *Xenopus* data. Recapitulating the leakiness observed in axolotl, both pre- and post-metamorphic animals displayed 1 kDa^+^ cells in the brain parenchyma (Fig. 5B-C’’), supporting that the differences observed in the BBB permeability threshold is not a trait directly related to metamorphosis. Different from axolotl, and in line with the results in *Xenopus* permeability, small amounts of tracer were also retained in the vasculature. These similarities were further validated by the Mander’s coefficients and no significant differences were observed between pre- and post-metamorphic *Pleurodeles* compared to either *Xenopus* or axolotl (Fig. 5D). Interestingly, the proportion of area covered by isolectin was slightly lower in *Pleurodeles* than in *Xenopus* and axolotl (Additional file 1: Fig. S1A), indicating a lower coverage of the brain parenchymal vascular system in this species compared to the other two.

### Claudin-5 expression pattern in the Amphibian blood–brain barrier

**Fig. 6:**
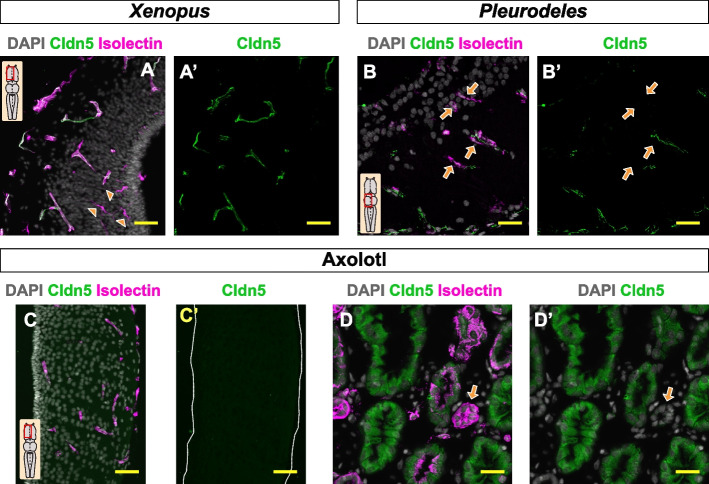
Claudin-5 expression pattern in *Xenopus*, *Pleurodeles* and axolotl. **A**-**C’’** Confocal images of a transversal section of the *Xenopus* telencephalon (**A**, **A’**), *Pleurodeles* midbrain (**B**, **B’**) and axolotl telencephalon (**C**, **C’**) stained with DAPI, isolectin and Claudin-5. The orange arrowheads marked isolectin.^+^ immune cells and orange arrows shown Claudin-5 negative blood vessels. **D**, **D’** Micrographs illustrating the axolotl testis labeled with DAPI, isolectin and Claudin-5. All images are full z-projections of a confocal stack. Scale bars = 50 µm. Abbreviations: Cldn5: Claudin-5

Previous studies have demonstrated that the disruption of the tight junction protein Claudin-5 is sufficient to increase paracellular leakage of molecules in a size-selective manner [29–31]. To test whether the leakage of the 1 kDa tracer observed in axolotl and *Pleurodeles* was based on differences in the tight junction configuration, we analyzed the expression pattern of Claudin-5 in all three amphibian species. *Xenopus* parenchymal blood vessels exhibited high levels of Claudin-5 expression covering all the blood vessels in the parenchyma (Fig. 6A, A’). The *Pleurodeles* brain displayed a diverse profile in the parenchymal vasculature, including vessels which were positive, weak and negative for Claudin-5 (Fig. 6B, B’). In contrast, the axolotl brain did not express any Claudin-5 in the parenchyma (Fig. 6C, C’)*.* As a positive control for the Claudin-5 antibody we used axolotl testis (Fig. 6D, D’). Taken together, the Claudin-5 expression levels correlated with our permeability analysis. Decrease or lack of the tight junction protein Claudin-5 might facilitate the leakage of the 1 kDa tracer.

## Discussion

CNS immune and metabolic privilege is a highly conserved feature during evolution. The brain is isolated from the periphery by different barriers that maintain the physiological homeostasis and ensure neuronal function. While both the BBB and the cerebrospinal fluid-brain barrier have been involved in immune surveillance, the metabolic supply is mainly provided through the BBB [1]. The tightness of the mammalian BBB has been classically assessed by the extravasation of exogenous tracers, since they have no biological relevance and should not be able to cross the barrier under physiological conditions [20]. The usage of tracers with known molecular size and the combination with different survival time points allowed us not only to assess the size permeability thresholds, but also to analyze which type of transport might be involved.

Our data revealed that the *Xenopus* BBB was impermeable to all the analyzed tracers (1, 3 and 70 kDa), recapitulating the BBB threshold previously described in mammals [20] and zebrafish [28]. On the contrary, axolotl exhibited a fast and systemic leakage of the 1 kDa tracer with no signs of EC transcellular uptake, supporting paracellular transport as the underlying mechanism. Passive leakage was also observed in pre- and post-metamorphic *Pleurodeles*, validating the size limit as a physiological trait not related to metamorphosis or exclusive the paedomorphic axolotl. Interestingly, and unlike axolotl, *Pleurodeles* also displayed partial retention of the 1 kDa tracer in the vasculature. This phenotype was half-way between the systemic leakiness observed in axolotl and the mammalian-like permeability found in *Xenopus*. Under physiological conditions, paracellular transport is severely restricted by tight junction protein complexes that seal the paracellular space between neighboring endothelial cells. Claudin-5 is the most enriched tight junction protein in the BBB, and it is essential to maintain the pore size selectivity [29–31]. Interestingly, the leakage of the 1 kDa tracer observed in axolotl correlated with the absence in the expression of Claudin-5. The partial leakage observed in *Pleurodeles* correlated with an incomplete coverage of Claudin 5 in the blood vessels, while Claudin 5 was found in all *Xenopus* vessels. Further validation of this hypothesis would require gain- and loss-of-function experiments.

Leakiness of the mammalian BBB has been related to systemic inflammation [32], metabolic dysfunction [33] and progression of neurodegenerative diseases [6–8]. Importantly, single cell analyses [34, 35], and behavioral [36] and regenerative [37] studies in salamanders showed no signs of systemic inflammation or neuronal dysfunction. These studies raise the question whether the passive leakage observed in salamanders might represent a metabolic and/or immunological advantage. Whether the permeability of BBB is related to the urodele CNS regenerative abilities is also an open question.

Particularly surprising was the increase in the rate of endocytic events in the axolotl vasculature in comparison to *Xenopus*. It is important to mention, that in none of our experimental paradigms the active transport or leakage of the larger tracers (3 and 70 kDa) were observed, indicating that the axolotl EC sorting mechanisms successfully blocked the transport of molecules without biological relevance for the brain. Therefore, and despite the leakiness observed for small molecules, the axolotl BBB displayed a selective and efficient barrier for larger molecules. The EM analysis revealed the ultrastructure of the axolotl EC luminal surface, showing high density of long membrane ruffles, that in turn generated large intracellular vesicles. Considering the ultrastructure [38] and the uptake of non-selective large cargo, we found that the axolotl ECs exhibited higher rates of (macro)pinocytosis. This endocytic pathway is nonspecific and nonsaturable, since it does not rely on any receptor or carrier, explaining how a synthetic molecule can cross the first barrier of the BBB. Macropinocytosis has been generally associated to nutrient sensing, antigen presentation and recycling of plasma proteins among other functions [38]. However, it is normally downregulated during development [39] and remains unknown how much of the metabolic supply relays on this type of transport. Despite being less efficient than the receptor-mediated transcytosis, it has been proposed that macropinocytosis might act as a non-canonical nutrient supply pathway, promoting metabolic flexibility and cellular resilience [40]. Zhulyn and collaborators have recently demonstrated that upon limb amputation, translation from pre-existing RNAs is rapidly activated via mTOR signaling pathway, promoting scarless regeneration in axolotl [41]. The presence of a dormant transcriptome supports the notion of a “ready-to-go” regenerative program that ensures a rapid and successful healing. However, protein synthesis comes at a high metabolic cost and amino acid transport at the time of injury is essential for wound closure [41]. Macropinocytosis is known not only to supply nutrients and amino acids via lysosomal degradation to support metabolism and macromolecular synthesis, but also to stimulate mTORC1 activity [42]. Our working hypothesis is that the increased macropinocytosis rate observed in axolotl ECs might provide a metabolic advantage during the regenerative process, rapidly increasing the energy supply, which potentially promotes cell survival and contributes to the scarless wound closure. On the other hand, the impermeable mammalian-type of BBB described in many vertebrates [32, 37], including *Xenopus*, might represent an obstacle for the scarless regenerative process [43, 44].

## Conclusions

Overall, our work highlights important structural and functional differences in the BBB in two groups of amphibians: salamanders and frogs. Considerations to the higher permeability observed in axolotl and *Pleurodeles* might be beneficial for targeted therapeutics, developing new drug delivery systems and for unravelling new mechanisms ameliorating the effects of BBB disruptions. In addition, understanding how salamanders regulate the BBB permeability in physiological and injury conditions might shed some light on the mechanisms underlying their extraordinary regenerative capacity.

## Methods

### Animals and experimental preparation

Pre-metamorphic larval *Xenopus laevis* (stage 49, stage 59–61 and stage 66; Nieuwkoop et al., 1994 [45]) and 5—7 cm (juvenile) *Ambystoma mexicanum* of either sex were obtained from the in-house animal breeding facility at the Biocenter-Martinsried of the Ludwig-Maximilians-University Munich. Both species were maintained in separate tanks with non-chlorinated water (17–19 °C) at a 12/12 light/dark cycle. The density of animals maintained in the study was 1.6 axolotl and 1.8 Xenopus per square meter. *Xenopus* were fed daily with spirulina algae and axolotl with daphnia and artemia.

At the desired developmental stage, animals were used for the studies in accordance with the “Principles of animal care” publication No. 86–23, revised 1985, of the National Institutes of Health and were carried out in accordance with the ARRIVE guidelines and regulations. Permission for the experiments was granted by the legally responsible governmental body of Upper Bavaria (Regierung von Oberbayern) under the license code ROB-55.2.2532.Vet_03-19–63. In addition, all experiments were performed in accordance with the relevant guidelines and regulations of the Ludwig-Maximilians-University Munich.

*Pleurodeles waltl* were bred and maintained in the aquatic facility in the Karolinska Institute. All of the procedures in this study related to animal handling, care and treatment were performed according to the guidelines approved by the Jordbruksverket/Sweden under the ethical permit numbers 18190–18 and 5723–2019. Late active larvae (Stage 53- 54) and post-metamorphic newts (Stage 56,2) were used for this study, stages according to Gallien and Durocher [46]. Pleurodeles were kept at a density of 0.5 animals per square meter and fed with artemia and grindal worms [47].

### Assessment of tracer retention in brain blood vessels

To assess the BBB permeability, 1 kDa Alexa Fluor 555 Cadaverine (Thermofisher; Germany), 3 kDa or 70 kDa dextran coupled to Tetramethylrhodamine (Thermofisher; Germany) were transcardially injected together with Isolectin GS-IB4 conjugated with Alexa 647 (Thermofisher; Germany). The animals were randomly allocated into the different experimental groups. As described previously [21], animals were anesthetized in 0.05% 3-aminobenzoic acid ethyl ester methanesulfonate at room temperature (MS-222; Pharmaq Ltd. UK) for 5—7 min. Thereafter, anesthetized animals were transferred to a Petri dish (Ø 5 cm) filled with ice cold Ringer solution (75 mM NaCl, 25 mM NaHCO_3_, 2 mM CaCl_2_, 2 mM KCl, 0.1 mM MgCl_2_, and 11 mM glucose, pH 7.4) containing 0.05% MS-222 and were mechanically secured onto a Sylgard-lined floor with the ventral side up. For *Pleurodeles*, injections were conducted by placing the deeply anesthetized animals on top of a Tricaine-soaked tissue. The skin and pericardial membrane were carefully excised to expose the beating heart (see [21]). Glass capillaries (outer diameter Ø 1 mm) were pulled on a P-87 Brown/Flaming electrode puller from borosilicate glass (Science Products; Germany). Capillaries were backfilled with different combinations of the dyes (10 µl volume), inserted into a micro-pipette holder, attached to a 3-axes, manually driven micro-manipulator (Bachhofer, Reutlingen, Germany) and connected to a pressure injector (FemtoJet, Eppendorf, Germany). The tip of the glass capillary was broken to ˜30 µm and the pressure was set such that each pulse corresponded to an injected volume of ˜0.1—0.2 μl. A 1:1 mix of 1.25 µg/µl Isolectin with 1 µg/µl Cadaverine (1kD) or 5 µg/µl 3 kDa dextran or 2.5 µg/µl 70 kDa dextran were injected. Pulses were triggered manually according to the heart beat rhythm until a volume of 5—8 μl was injected for both axolotl and pre-metamorphic *Xenopus*. After post-injection survival times (the period of time from the dye injection to the excision of the heart) in deep anesthesia for 20 or 60 min, animals were decapitated and the skull was dorsally and ventrally opened by removing the skin and the cartilage to completely expose the brain for proper fixation of the tissue with 4% paraformaldehyde (PFA) for 24 h at 4 °C. For the experiments in *Pleurodeles*, a 1:1 mix of 1 µg/µl Cadaverine (1kD) and 1–2.5 µg/µl Isolectin was injected through the base of the aorta in deeply anesthetized animals. A volume of 1.5, 2.75 or 7.5 µl was injected in animals of stage 53, 54 and 56 respectively. After injection, animals were placed back in anesthesia for 20 min followed by decapitation and fixation in 4% PFA.

### Tissue clearance

Following fixation, whole mount preparations consisting of brain and spinal cord kept in place inside the open skulls, were washed in PBS (3 × for 20 min) prior to the tissue clearing process with the uDisco protocol [48]. Whole mount preparations were step-wise dehydrated for 4 h in increasing concentrations of butanol (30, 50, 70, 80, 90, 96, 100%). While the axolotl and *Pleurodeles* tissue was incubated in dichloromethane for 40—60 min prior to the clearing process, *Xenopus* tissue did not require this additional pretreatment. While red and far-red fluorochromes were not affected by our uDisco protocol; green fluorophores were non-discernible from green autofluorescence of fixed tissue especially after dichloromethane treatment. The tissue was then incubated in a 1:2 solution of benzylbenzoate and benzylalcohol, mixed 15:1 with diphenylether for 48 h. The cleared brains along with the spinal cords were subsequently extracted from the skull and mounted in the clearing solution using custom-built metal spacers. Whole mounts of the CNS tissue were imaged using a Leica SP5-2 confocal microscope. Between 50–100 optical sections of 2 µm thickness per brain area were obtained (for methodological adaptations of the protocol for *Xenopus* see also [49]).

### Evaluation of dye uptake into endothelial cells

Juvenile axolotls were transcardially injected (70 kDa dye together with isolectin) according to the previously described protocol. After a post-injection survival time of 20 min, animals were perfused with Ringer solution (specified above) over a period of 5 min. The brain and spinal cord were extracted immediately afterwards and the complete removal of blood and dye by the perfusion was validated under a fluorescence stereo-microscope (SZX16, Olympus, Japan). Thereafter, the tissue was fixed with 4% PFA for 24 h at 4 °C. After washing with PBS (3 × for 20 min), brains were embedded in 3% agarose dissolved in PBS. Tissue blocks were cryoprotected for at least 3 days in a 30% sucrose solution at 4 °C. 30 µm thick coronal sections were cut on a cryostat (Leica, Germany) and mounted on gelatin-coated slides (VWR, Belgium) with Aquamont (Aqua-Poly/ Mount, Polysciences, Warrington, PA).

### Quantification and data analysis

Optical sections, obtained from cleared whole mount preparations were automatically analyzed and quantified using the Fiji plug-in JACOP [50]. A pixel-wise co-occurrence of fluorescence signals of the selected tracer and the blood vessel marker isolectin was measured using Manders’ coefficients. This procedure yielded two coefficients: M_isolectin_ (co-occurrence of isolectin with a particular dye) and M_dye_ (co-occurrence of the selected dye with isolectin). All areas of the CNS (telencephalon, midbrain, hindbrain and spinal cord) were used for the quantitative analyses. A total of 2 images obtained from two different CNS areas were used to obtain the Manders’ coefficients per animal. The percentage of isolectin-covered area was calculated for each animal using two confocal images obtained from cleared brains. Single-channel immunofluorescent images were converted to black and white, then every image in the Z-stack was thresholded and the extent of the stained area was measured using NIH ImageJ software.

Statistical analysis and plotting were performed in Prism 9 (GraphPad Software Inc.) and the data were tested for normality by the Shapiro–Wilk normality test. If both normality and equal group variances assumption were met Student´s t-test for single comparisons and One-way ANOVA for multiple group design were used. Welch´s t-test was selected when the groups had unequal variances. For non-parametric tests simple linear regression or Kruskal–Wallis test for multiple group design were used. Population data is reported as mean ± standard error of the mean (SEM). Statistical tests and respective details can also be found in the relevant figure legends and/or corresponding result sections. N-values used in statistical tests represent number of animals and a minimum of three independent experiments were performed. Figures were assembled using Affinity Designer 1.10.5.1342 (Serif, UK).

### Electron microscopy and 3D reconstruction

A juvenile axolotl and a pre-metamorphic *Xenopus* were anaesthetized as previously described and perfused with Ringer’s solution, followed by 2.5% glutaraldehyde with 2% PFA in cacodylate buffer. Subsequently, the hindbrains were isolated, cut in ca. 1 mm pieces and postfixed in the same fixative by immersion overnight at 4 °C, followed by a high-contrast staining for block-face EM (protocol modified from Hua et al. [51]). In brief, the tissue blocks were immersed in buffered 2% OsO_4_, 1.5% ferrocyanide, saturated thiocarbohydrazide, unbuffered 2% OsO_4_, 1% uranyl acetate, and lead aspartate with intermittent washing steps. Thereafter, the brain tissue was dehydrated in a graded ethanol series, transferred to dried acetone and embedded in hard-plus resin 812 (EM sciences). One surface of a resin block was trimmed to remove empty resin and mounted upside down on an aluminum stub with conductive glue. The remaining block was trimmed into the shape of a cube (300 µm edges) with no empty resin on all sides and coated with gold in a sputter coater. The samples were sectioned and imaged with an Apreo VS block-face scanning electron microscope (Thermo Fisher) at 2 kV in high vacuum mode, pixel dwell time 3 µs. Axolotl: 1400 planes, slice spacing 40 nm, scanning area 102 × 102 µm @ 10 nm/px. For 3D reconstruction, a subvolume of 1800 × 5900 × 411 px was selected, containing a short section of a capillary pair. *Xenopus*: 1300 planes, slice spacing 35 nm, scanning area 30 × 30 µm @ 5 nm/px. For 3D reconstruction, a subvolume of 5300 × 5800 × 458 px was selected. In every species the inner surface of the endothelial cells of one capillary was segmented and surface rendered using Amira 5.6 software.

## Supplementary Information

Additional file 1: Figure S1 Quantifications of the isolectin^+^ staining and the tracer retention in the Amphibian CNS.Dot-plot illustrating the proportion of isolectin covered area in the *Xenopus*, axolotland pre-and post-metamorphic *Pleurodeles* brain. Data are shown as mean ± SEM and statistical analysis is based on Kruskall-Wallis testand Dunn’s multiple comparisons test.Dot-plot depicting the Manders’ coefficients of all the analyzed tracers in *Xenopus* and axolotl. The data were color-coded according to the tracers. Each data point represents one animal. Data are shown as mean ± SEM and statistical analysis is based on simple linear regression. Abbreviations: n.s: not significant; Pre: pre-metamorphic; Post: post-metamorphic. Figure S2 Permeability analysis of the 70 kDa tracer using a long survival protocol.Scheme of the experimental design of the 60 min survival protocol.Images depicting the retention pattern of the 70 kDa tracer in the axolotl and the *Xenopus* hindbrain. Low retention areas are indicated with orange arrowheads. Insets indicate the area of the brain. All images are full z-projections of a confocal stack. Scale bars = 100 µm.Dot-plot analyzing the M_isolectin_ coefficients of the 70 kDa tracer in the axolotl and *Xenopus* CNS after 20and 60mins survival. Each data point represents one animal. Data are shown as mean ± SEM. Abbreviations: Ax: axolotl; Xe: *Xenopus*. Figure S3 Details of the EM images from the axolotl and *Xenopus* brain.Macropinocytosis, plane spacing 80 nm; red asterisk: structure engulfing some EC lumen; white asterisk: macropinosome with flaky material.Micropinocytosis, plane spacing 40 nm; arrow: micropinocytosis in statu nascendi; white arrowhead: small vesicles.Extracellular vesicles containing flaky electron dense material in the *Xenopus* luminal space.Micropinosomes in *Xenopus* ECs. Plane spacing in C and D 35 nm. Scale bars in A, B = 500 nm; in C, D = 1 µm. Figure S4 Comparative analysis of the permeability threshold in the *Xenopus* and axolotl BBB.Dot-plots showing the M_isolectin_ coefficients of all injected tracers following the 20 min survival protocol in *Xenopus*and axolotl. The data were color-coded according to the molecular size. Each data point represents one animal. Data are shown as mean ± SEM and statistical analysis is based on One-way ANOVA.Experimental workflow to analyze the 1 kDa leakage observed in the axolotl CNS.Confocal image of a transversal section of the axolotl telencephalon injected with 1 kDa tracer and labeled with DAPI.Images depicting the retention pattern of the 1 kDa tracer in *Xenopus* at stage 49and stage 66. Inset indicate the area of the brain. The images are full z-projections of a confocal stack. Scale bars in D, D’, D’’ = 50 µm; in E, E’, F, F’’ = 100 µm. Figure S5 Comparative analysis of the permeability threshold of the injected tracers in all analyzed species.Dot-plots depicting the Manders’ coefficients of the Xenopus, Axolotl and *Pleurodeles* BBB. In A, data was colored-coded by tracer, 1 kDa. The yellow area was further analyzed in B.The Manders’ coefficients of the 1 kDa injected *Xenopus*, axolotl, pre-and post-metamorphic*Pleurodeles* were plotted. Each data point represents one animal.

## Abbreviations

Axo: Axolotl
Ba: Basilar artery
BBB: Blood–brain barrier
CNS: Central nervous system
Cldn5: Claudin-5
ECs: Endothelial cells
ltv: Lateral telencephalic vein
Moa: Medial olfactory artery
n.s: Not significant
PFA: Paraformaldehyde
Pb: Posterior branch of cerebral carotid
Poa: Preoptic artery
Post: Post-metamorphic
Pre: Pre-metamorphic
SEM: Standard error of the mean
V: Ventricle
Xe: Xenopus

## References

[1] Knox EG, Aburto MR, Clarke G, Cryan JF, O’Driscoll CM. The blood-brain barrier in aging and neurodegeneration. Mol Psychiatry. 2022;27:2659–73. 35361905 10.1038/s41380-022-01511-z PMC9156404

[2] Haddad-Tóvolli R, Dragano NR, Ramalho AF, Velloso LA. Development and function of the blood-brain barrier in the context of metabolic control. Front Neurosci. 2017;11:224. 28484368 10.3389/fnins.2017.00224 PMC5399017

[3] Mink JW, Blumenschine RJ, Adams DB. Ratio of central nervous system to body metabolism in vertebrates: its constancy and functional basis. Am J Physiol Regul Intgr Comp Physiol. 1981;241(3):R203–12. 10.1152/ajpregu.1981.241.3.R203 7282965

[4] Ames A. CNS energy metabolism as related to function. Brain Res Rev. 2000;34(1–2):42–68. 11086186 10.1016/s0165-0173(00)00038-2

[5] Schläger C, Körner H, Krueger M, Vidoli S, Haberl M, Mielke D, et al. Effector T-cell trafficking between the leptomeninges and the cerebrospinal fluid. Nature. 2016;530(7590):349–53. 26863192 10.1038/nature16939

[6] Greene C, Hanley N, Reschke CR, Reddy A, Mäe MA, Connolly R, et al. Microvascular stabilization via blood-brain barrier regulation prevents seizure activity. Nat Commun. 2022;13:2003. 35422069 10.1038/s41467-022-29657-y PMC9010415

[7] Senatorov VV Jr, Friedman AR, Milikovsky DZ, Ofer J, Saar-Ashkenazy R, Charbash A, et al. Blood-brain barrier dysfunction in aging induces hyperactivation of TGFβ signaling and chronic yet reversible neural dysfunction. Sci Transl Med. 2019;11(521). 10.1126/scitranslmed.aaw8283 31801886

[8] Profaci CP, Munji RN, Pulido RS, Daneman R. The blood-brain barrier in health and disease: Important unanswered questions. J Exp Med. 2020;217(4). 10.1084/jem.20190062 PMC7144528 32211826

[9] Dunton AD, Göpel T, Ho DH , Burggren W. Form and Function of the Vertebrate and Invertebrate Blood-Brain Barriers. Int J Mol Sci. 2021; 9;22(22):12111 10.3390/ijms222212111 PMC8618301 34829989

[10] Schaeffer S, Iadecola C. Revisiting the neurovascular unit. Nat Neurosci. 2021;24(9):1198–209. 34354283 10.1038/s41593-021-00904-7 PMC9462551

[11] Fenstermacher J, Kaye T. Drug, “diffusion” within the brain. Ann N Y Acad Sci. 1988;531:29–39. 3382143 10.1111/j.1749-6632.1988.tb31809.x

[12] Sedlakova R, Shivers RR, Del Maestro RF. Ultrastructure of the blood-brain barrier in the rabbit. J Submicrosc Cytol Pathol. 1999;31(1):149–61. 10363362

[13] Kniesel U, Wolburg H. Tight junctions of the blood-brain barrier. Cell Mol Neurobiol. 2000;20:57–76. 10690502 10.1023/A:1006995910836 PMC11537529

[14] Vanlandewijck M, He L, Mäe MA, Andrae J, Ando K, Del Gaudio F, et al. A molecular atlas of cell types and zonation in the brain vasculature. Nature. 2018;554(7693):475–80. 29443965 10.1038/nature25739

[15] Heithoff BP, George KK, Phares AN, Zuidhoek IA, Munoz-Ballester C, Robel S. Astrocytes are necessary for blood-brain barrier maintenance in the adult mouse brain. Glia. 2021;69(2):436–72. 32955153 10.1002/glia.23908 PMC7736206

[16] Liu CY, Yang Y, Ju WN, Wang X, Zhang HL. Emerging roles of astrocytes in neuro-vascular unit and the tripartite synapse with emphasis on reactive gliosis in the context of Alzheimer’s disease. Front Cell Neurosci. 2018;12:193. 30042661 10.3389/fncel.2018.00193 PMC6048287

[17] Falcone C. Evolution of astrocytes: From invertebrates to vertebrates. Front Cell Dev Biol. 2022;10: 931311. 36046339 10.3389/fcell.2022.931311 PMC9423676

[18] Joven A, Wang H, Pinheiro T, Hameed LS, Belnoue L, Simon A. Cellular basis of brain maturation and acquisition of complex behaviors in salamanders. Development. 2018 8;145(1):dev160051. 10.1242/dev.160051 29217751

[19] Joven A, Simon A. Homeostatic and regenerative neurogenesis in salamanders. Prog Neurobiol. 2018;170:81–98. 29654836 10.1016/j.pneurobio.2018.04.006

[20] Yanagida K, Liu CH, Faraco G, Galvani S, Smith HK, Burg N, et al. Size-selective opening of the blood–brain barrier by targeting endothelial sphingosine 1–phosphate receptor 1. PNAS. 2017;114(17):4531–6. 28396408 10.1073/pnas.1618659114 PMC5410849

[21] Özugur S, Chávez MN, Sanchez-Gonzalez R, Kunz L, Nickelsen J, Straka H. Transcardial injection and vascular distribution of microalgae in Xenopus laevis as means to supply the brain with photosynthetic oxygen. STAR Protoc. 2022;3(2):101250. 35313711 10.1016/j.xpro.2022.101250 PMC8933832

[22] Wälchli T, Mateos JM, Weinman O, Babic D, Regli L, Hoerstrup SP, et al. Quantitative assessment of angiogenesis, perfused blood vessels, and endothelial tip cells in the postnatal mouse brain. Nat Protoc. 2015;10:53–74. 10.1038/nprot.2015.002 25502884

[23] Lametschwandtner A, Minnich B. Microvascular anatomy of the brain of the adult pipid frog, Xenopus laevis (Daudin): A scanning electron microscopic study of vascular corrosion casts. J Morphol. 2018;279(7):950–69. 10.1002/jmor.20824 PMC6718010 29693258

[24] Kadry H, Noorani B, Cucullo L. A blood–brain barrier overview on structure, function, impairment, and biomarkers of integrity. Fluids Barriers CNS. 2020;17:1–24. 10.1186/s12987-020-00230-3 PMC7672931 33208141

[25] Yang AC, Stevens MY, Chen MB, Lee DP, Stähli D, Gate D, et al. Physiological blood-brain transport is impaired with age by a shift in transcytosis. Nature. 2020;583:425–30. 10.1038/s41586-020-2453-z PMC8331074 32612231

[26] Villaseñor R, Lampe J, Schwaninger M, Collin L. Intracellular transport and regulation of transcytosis across the blood–brain barrier. Cell Mol Life Sci. 2019;76:1081–92. 10.1007/s00018-018-2982-x PMC6513804 30523362

[27] De Jesus Andino F, Jones L, Maggirwar SB, Robert J. Frog Virus 3 dissemination in the brain of tadpoles, but not in adult Xenopus, involves blood-brain barrier dysfunction. Sci Rep. 2016;6:22508. 10.1038/srep22508 PMC4773881 26931458

[28] O'Brown NM, Megason SG, Gu C. Suppression of transcytosis regulates zebrafish blood-brain barrier function. Elife. 2019;8:e47326. 10.7554/eLife.47326 PMC6726461 31429822

[29] Nitta T, Hata M, Gotoh S, Seo Y, Sasaki H, Hashimoto N, Furuse M, Tsukita S. Size-selective loosening of the blood-brain barrier in claudin-5-deficient mice. J Cell Biol. 2003;161(3):653–60. 10.1083/jcb.200302070 PMC2172943 12743111

[30] Campbell M, Kiang AS, Kenna PF, Kerskens C, Blau C, O'Dwyer L, Tivnan A, Kelly JA, Brankin B, Farrar GJ, Humphries P. RNAi-mediated reversible opening of the blood-brain barrier: J Gene Med. 2008;10(8):930–47. 10.1002/jgm.1211 18509865

[31] Vazquez-Liebanas E, Mocci G, Li W, Lavina B, Reddy A, O’Connor C, Hudson N, Elbeck Z, Nikoloudis I, Gaengel K, Vanlandewijck M, Campbell M, Betsholtz C, Mae MA. Mosaic deletion of claudin-5 reveals rapid non-cell-autonomous consequences of blood-brain barrier leakage. Cell Reports. 2024;43:113911. 10.1016/j.celrep.2024.113911 38446668

[32] Takata F, Nakagawa S, Matsumoto J, Dohgu S. Blood-brain barrier dysfunction amplifies the development of neuroinflammation: understanding of cellular events in brain microvascular endothelial cells for prevention and treatment of BBB dysfunction. Front Cell Neurosci. 2021;15:661838. 10.3389/fncel.2021.661838 PMC8475767 34588955

[33] Segarra M, Aburto MR, Acker-Palmer A. Blood–brain barrier dynamics to maintain brain homeostasis. Trends Neurosci. 2021;44(5):393–405. 10.1016/j.tins.2020.12.002 33423792

[34] Woych J, Ortega Gurrola A, Deryckere A, Jaeger ECB, Gumnit E, Merello G, Gu J, Joven Araus A, Leigh ND, Yun M, Simon A, Tosches MA. Cell-type profiling in salamanders identifies innovations in vertebrate forebrain evolution. Science. 2022;377(6610):eabp9186. 10.1126/science.abp9186 PMC10024926 36048957

[35] Lust K, Maynard A, Gomes T, Fleck JS, Camp JG, Tanaka EM, Treutlein B. Single-cell analyses of axolotl telencephalon organization, neurogenesis, and regeneration. Science. 2022;377(6610):eabp9262. 10.1126/science.abp9262 36048956

[36] Knorr AG, Gravot CM, Gordy C, Glasauer S, Straka H. I Spy with My Little Eye: A Simple Behavioral Assay to Test Color Sensitivity on Digital Displays. Biol Open. 2018;7(10):bio035725. 10.1242/bio.035725 PMC6215414 30127095

[37] Amamoto R, Huerta VG, Takahashi E, Dai G, Grant AK, Fu Z, Arlotta P. Adult axolotls can regenerate original neuronal diversity in response to brain injury. Elife. 2016;5:e13998. 10.7554/eLife.13998 PMC4861602 27156560

[38] Lin XP, Mintern JD, Gleeson PA. Macropinocytosis in different cell types: similarities and differences. Membranes. 2020;10(8):177. 10.3390/membranes10080177 PMC7463864 32756454

[39] Ben-Zvi A, Lacoste B, Kur E, Andreone BJ, Mayshar Y, Yan H, Gu C. Mfsd2a is critical for the formation and function of the blood–brain barrier. Nature. 2014;509(7501):507–11. 10.1038/nature13324 PMC4134871 24828040

[40] Recouvreux MV, Commisso C. Macropinocytosis: a metabolic adaptation to nutrient stress in cancer. Front Endocrinol. 2017;8:261. 10.3389/fendo.2017.00261 PMC5649207 29085336

[41] Zhulyn O, Rosenblatt HD, Shokat L, Dai S, Kuzuoglu-Öztürk D, Zhang Z, et al. Evolutionarily divergent mTOR remodels translatome for tissue regeneration. Nature. 2023;620:163–71. 10.1038/s41586-023-06365-1 PMC11181899 37495694

[42] Yoshida S, Pacitto R, Inoki K, Swanson J. Macropinocytosis, mTORC1 and cellular growth control. Cell Mol Life Sci. 2018;75:1227–39. 10.1007/s00018-017-2710-y PMC5843684 29119228

[43] Dimou L, Götz M. Glial cells as progenitors and stem cells: new roles in the healthy and diseased brain. Physiol Rev. 2014;94(3):709–37. 10.1152/physrev.00036.2013 24987003

[44] Sanchez-Gonzalez R, Koupourtidou C, Lepko T, Zambusi AT, Novoselc KT, Durovic T, et al. Innate immune pathways promote oligodendrocyte progenitor cell recruitment to the injury site in adult zebrafish brain. Cells. 2022;11(3):520. 10.3390/cells11030520 PMC8834209 35159329

[45] Nieuwkoop PD, Faber J. Normal Table of Xenopus laevis (Daudin): a Systematical and Chronological Survey of the Development from the Fertilized Egg Till the end of Metamorphosis. New York: Garland Pub; 1994.

[46] Gallien L, Durocher M. Table chronologique du développement chez Pleurodeles waltlii Michah. Bull Biol Fr Belg. 1957;91:97–114.

[47] Joven A, Kirkham M, Simon A. Husbandry of Spanish ribbed newts (Pleurodeles waltl). Methods Mol Biol. 2015;1290:47–70. 10.1007/978-1-4939-2495-0_4 25740476

[48] Pan C, Cai R, Quacquarelli FP, Ghasemigharagoz A, Lourbopoulos A, Matryba P, et al. Shrinkage-mediated imaging of entire organs and organisms using uDISCO. Nat Methods. 2016;13:859–67. 10.1038/nmeth.3964 27548807

[49] Forsthofer M, Straka H. Homeostatic plasticity of eye movement performance in Xenopus tadpoles following prolonged visual image motion stimulation. J Neurol. 2023;270:57–70. 10.1007/s00415-022-11311-8 PMC9813097 35947153

[50] Bolte S, Cordelières FP. A guided tour into subcellular colocalization analysis in light microscopy. J Microsc. 2006;224(3):213–32. 10.1111/j.1365-2818.2006.01706.x 17210054

[51] Hua Y, Laserstein P, Helmstaedter M. Large-volume en-bloc staining for electron microscopy-based connectomics. Nat Commun. 2015;6:7923. 10.1038/ncomms8923 PMC4532871 26235643

