# Supplementary material for: Species-specific blood–brain barrier permeability in amphibians
Source: BMC Biol. 2025 Feb 11;23:43. doi: 10.1186/s12915-025-02145-7 (PMC11817546; doi:10.1186/s12915-025-02145-7)
Supplement: Supplementary file 1 — Additional file 1: Figure S1 Quantifications of the isolectin+ staining and the tracer retention in the Amphibian CNS.Dot-plot illustrating the proportion of isolectin covered area in the Xenopus, axolotland pre-and post-metamorphic Pleurodeles brain. Data are shown as mean ± SEM and statistical analysis is based on Kruskall-Wallis testand Dunn’s multiple comparisons test.Dot-plot depicting the Manders’ coefficients of all the analyzed tracers in Xenopus and axolotl. The data were color-coded according to the tracers. Each data point represents one animal. Data are shown as mean ± SEM and statistical analysis is based on simple linear regression. Abbreviations: n.s: not significant; Pre: pre-metamorphic; Post: post-metamorphic. Figure S2 Permeability analysis of the 70 kDa tracer using a long survival protocol.Scheme of the experimental design of the 60 min survival protocol.Images depicting the retention pattern of the 70 kDa tracer in the axolotl and the Xenopus hindbrain. Low retention areas are indicated with orange arrowheads. Insets indicate the area of the brain. All images are full z-projections of a confocal stack. Scale bars = 100 µm.Dot-plot analyzing the Misolectin coefficients of the 70 kDa tracer in the axolotl and Xenopus CNS after 20and 60mins survival. Each data point represents one animal. Data are shown as mean ± SEM. Abbreviations: Ax: axolotl; Xe: Xenopus. Figure S3 Details of the EM images from the axolotl and Xenopus brain.Macropinocytosis, plane spacing 80 nm; red asterisk: structure engulfing some EC lumen; white asterisk: macropinosome with flaky material.Micropinocytosis, plane spacing 40 nm; arrow: micropinocytosis in statu nascendi; white arrowhead: small vesicles.Extracellular vesicles containing flaky electron dense material in the Xenopus luminal space.Micropinosomes in Xenopus ECs. Plane spacing in C and D 35 nm. Scale bars in A, B = 500 nm; in C, D = 1 µm. Figure S4 Comparative analysis of the permeability threshold in the Xenopus a [file 12915_2025_2145_MOESM1_ESM.pdf]

A

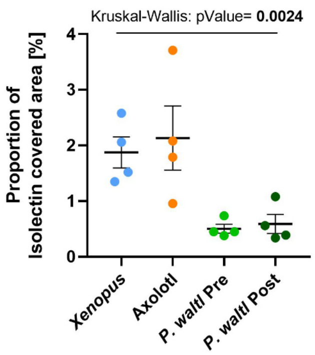

Dunn's multiple comparisons test

Species \ Species	*Xenopus*	*P. waltl* Pre	*P. waltl* Post	Axolotl
Xenopus		n.s	n.s	n.s
P. waltl Pre	n.s		n.s	n.s
P. waltl Post	n.s	n.s		n.s
Axolotl	n.s	n.s	n.s	

B

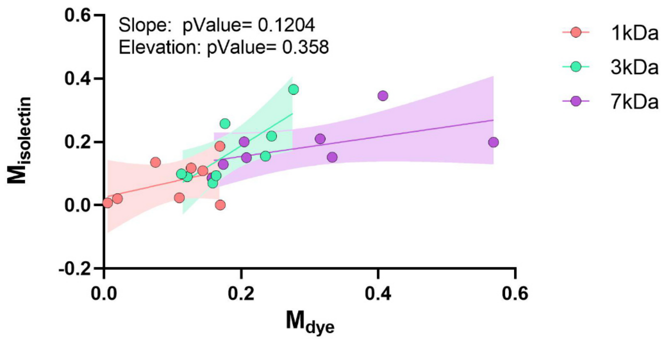

Additional file 1: Fig. S1

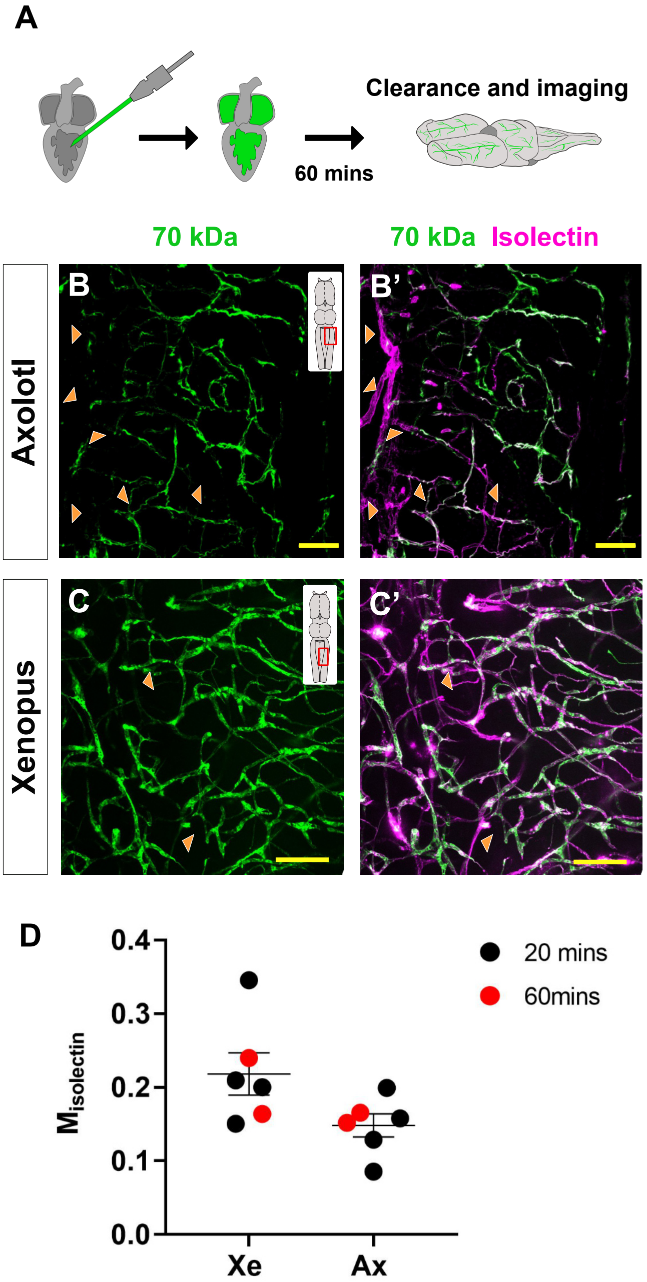

Additional file 1: Fig. S2

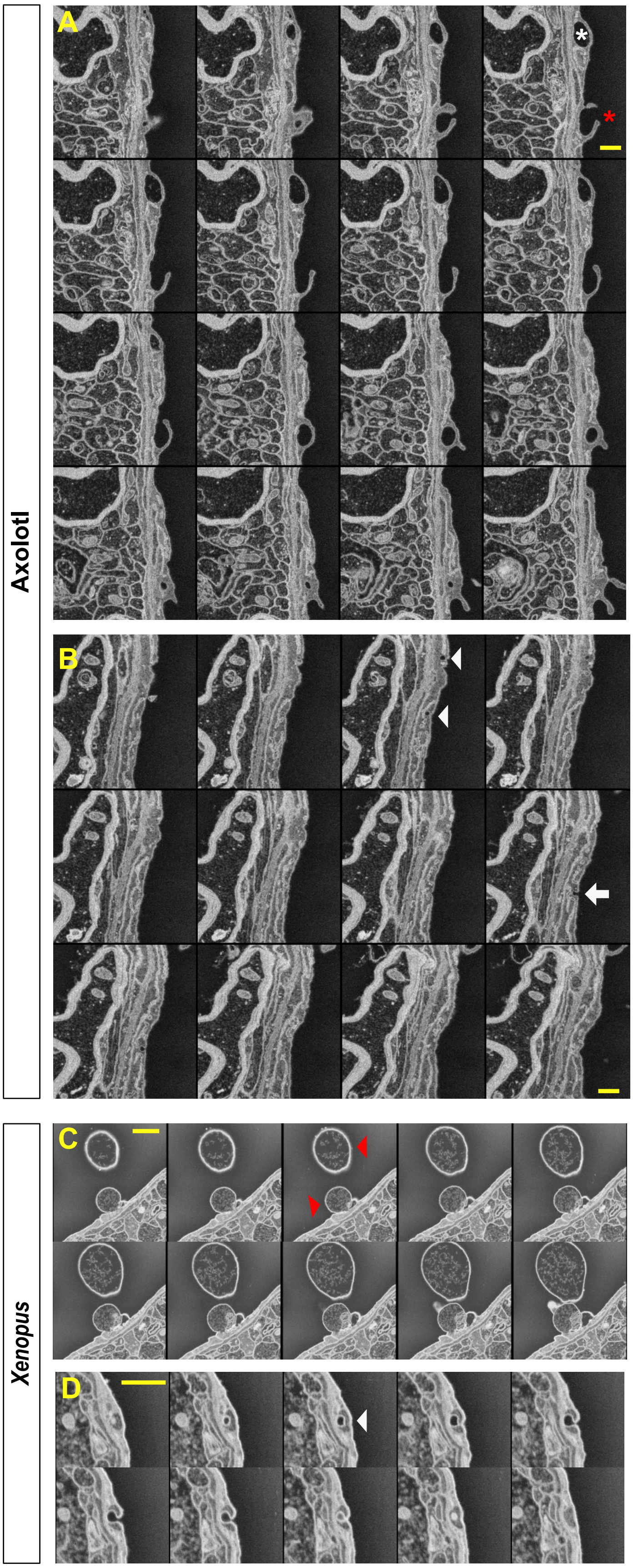

Additional file 1: Fig. S3

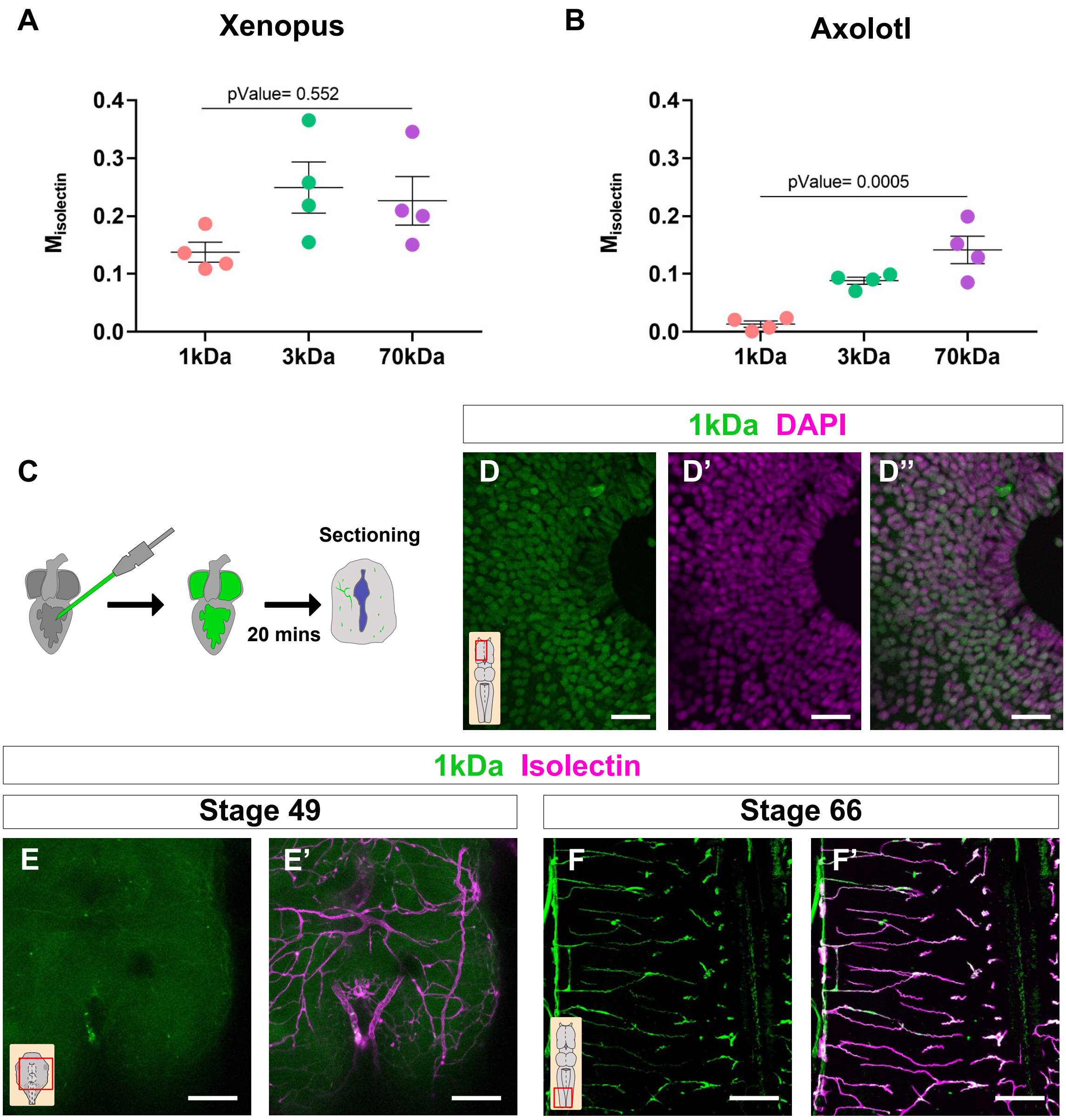

Additional file 1: Fig. S4

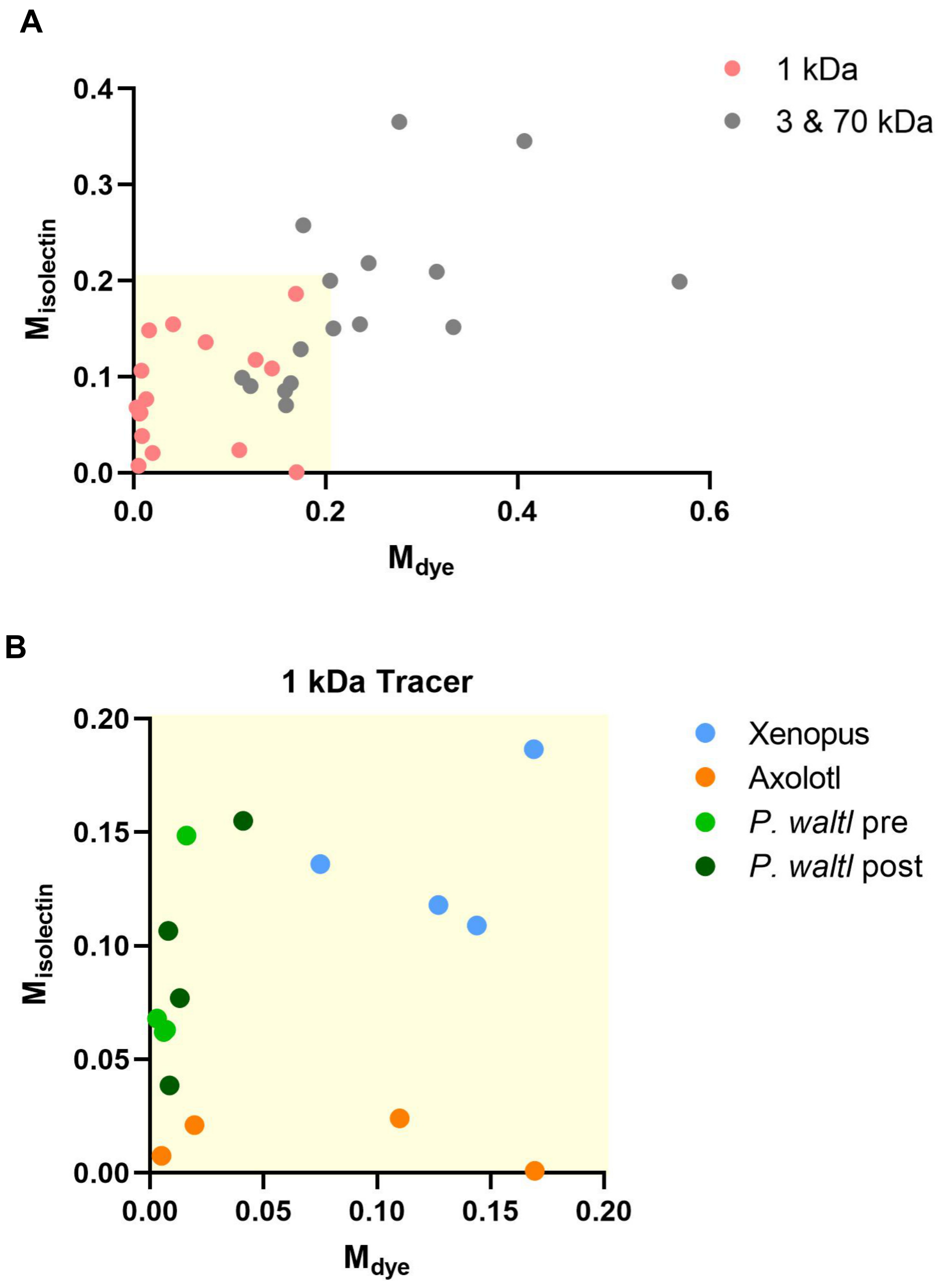

Additional file 1: Fig. S5
